# “What does it depend on?”: Perceptions of safety related to firearms in homes and neighborhoods

**DOI:** 10.1371/journal.pone.0261038

**Published:** 2021-12-29

**Authors:** Rocco Pallin, Garen J. Wintemute, Nicole Kravitz-Wirtz

**Affiliations:** 1 Violence Prevention Research Program, Department of Emergency Medicine, University of California Davis School of Medicine, Sacramento, California, United States of America; 2 University of California Firearm Violence Research Center, Sacramento, California, United States of America; Monash University, AUSTRALIA

## Abstract

**Background:**

Though research has established that firearms in the home increase risk for injury and death, a substantial number of Americans, especially gun owners, believe that guns make their homes safer. More than half of gun owners in a nationally-representative survey said “it depends” when asked whether guns make their homes safer or more dangerous, but little is known about the factors that affect perceived safety.

**Objective:**

To determine whether the relationship between the presence of firearms and perceived home or neighborhood safety is fixed or depends on additional factors and to identify the additional factors on which it depends.

**Methods:**

A mixed-methods cross-sectional analysis of the 2018 state-representative California Safety and Wellbeing Survey (n = 2558, completion rate 49%), including calculation of weighted proportions and qualitative analysis of write-in responses.

**Findings:**

One in six respondents (17.2%, 95% CI 14.9% to 19.7%) reported “it depends” when asked whether a gun in their home made the home a safer or more dangerous place to be (“the home scenario”). One in six (16.6%, 95% CI 14.3% to 19.2%) reported “it depends” when asked whether the neighborhood would be safer if all neighbors had guns in the home (“the neighborhood scenario”). For the home scenario, 28.3% (95% CI 21.9% to 35.7%) cited firearm owner characteristics (e.g., training and proficiency, temperament, and mental health), 28.4% (95% CI 22.3% to 35.5%) cited firearm storage and access, and 28.0% (95% CI 21.5% to 35.7%) cited intended use for guns as factors affecting perceived safety. For the neighborhood scenario, respondents overwhelmingly cited gun owner characteristics (72.1%, 95% CI 63.4% to 79.3%). Factors on which “it depends” varied by gun ownership status.

**Conclusion:**

Perceived safety when firearms are in the home depends on numerous factors. Understanding these factors may inform tailored, targeted messaging and interventions for firearm injury prevention.

## Background

Substantial epidemiological evidence suggests that guns in the home increase the risk of injury for everyone living there [1, 2]. Nonetheless, a significant and growing share of the United States population report that guns make homes safer: approximately three in five Americans say a gun in the home makes it safer, an increase from 35% in 2000 [3, 4]. Gun owners most often say guns make their homes safer when compared with non-owners [5], and research suggests owners with this belief less frequently store firearms locked up and unloaded, the safest storage method [6]. Related survey research also finds that only 15% of American adults agree with the statement that firearms in the home increase the risk of suicide, including just 6% of firearm owners, despite evidence to the contrary [7].

Past studies on firearm risk perceptions have primarily examined categorical self-reports that guns make homes either safer or more dangerous. What has received less attention are the perceptions of the individuals who do not endorse either sentiment unconditionally. The 2019 National Firearms Survey (NFS) found 56% of gun owners said “it depends” when asked whether guns make their homes safer or more dangerous, up from 40% in the 2015 NFS [6, 8]. Further, polling research finds about half of non-gun owners (and 23% of gun owners) neither agreed nor disagreed when asked whether guns in their homes make them safer [5]. Little is known about the factors that affect perceived safety and whether they vary by gun ownership status.

This mixed-methods study extends prior work using a state-representative survey of 2,558 California adults. We estimate the prevalence of perceived safety and dangerousness associated with having firearms in respondents’ own homes, as well as all homes in their neighborhoods, as past research documents that people tend to underrate their own vulnerability to risk while judging others as more susceptible [9, 10]. We then present qualitative analysis of the write-in responses expressed by those who said that whether guns make homes safer or more dangerous depended on one or more factors.

These findings may help in developing and implementing tailored firearm injury prevention strategies by yielding insight into the characteristics and conditions on which perceptions about firearm-related risk are predicated. Further, state-level data on firearm-related risk perceptions has, until this point, not been available. This is a critical gap in knowledge, as most firearm injury prevention strategies are undertaken at the state level and rates of firearm ownership and firearm-related death and injury vary markedly from state to state.

## Methods

### Study sample

We analyzed data from the 2018 California Safety and Wellbeing Survey (CSaWS). CSaWS is a probability-based Internet survey designed by the authors and administered in fall 2018 by the survey research firm Ipsos, which maintains KnowledgePanel, an online respondent panel of more than 60,000 adults nationwide [11]. KnowledgePanel has been used widely for health and injury-related research [12, 13]. Members are recruited randomly and continuously, using probability-based sampling of the US Postal Service’s Delivery Sequence File. Further details on KnowledgePanel and the sampling frame have been published previously [14].

All members of KnowledgePanel who were residents of California, except those currently residing in an institutional setting, were eligible to participate in CSaWS; 5,232 panel members received emailed invitations to complete the survey. Non-responders received automatic reminder emails 3, 7, 12, and 19 days after the initial invitation. No incentives specific to this study were provided to respondents. The survey was available in both English and Spanish.

CSaWS was approved by the Institutional Review Board at the University of California Davis. Before beginning the online survey, participants read an electronic statement on informed consent. The process of survey initiation constituted their consent to participate. This study followed the American Association for Public Opinion Research (AAPOR) reporting guideline.

### Measures

We asked all respondents two questions to ascertain their membership in one of three “firearm ownership status” groups: owners of firearms (“owners”), those living in households with firearms but who do not personally own firearms (“non-owners in gun households”), and those not living in households with firearms (“non-owners in households without guns”). Respondents who reported personally owning firearms were also asked about types and quantities of firearms owned, reasons for ownership, and storage practices; these results have been published previously [14].

We asked all respondents about perceptions of safety and dangerousness related to firearms in their homes and all homes in their neighborhoods: 1) “Does/would having a gun at your home make it a safer place to be, or a more dangerous place to be?” (“the home scenario”), and 2) “If everyone in your neighborhood had guns at home, would that make your neighborhood a safer place to be, or a more dangerous place to be?” (“the neighborhood scenario”). Respondents could choose “safer,” “more dangerous,” “it depends,” or “don’t know.” If a respondent said “it depends” for either question, they were then asked, “What does it depend on?” and given an opportunity to specify responses in an open-text field.

We obtained respondent age, race/ethnicity, sex, and rurality of residence, which were collected at enrollment in the panel and updated annually, directly from Ipsos.

### Analysis

We used a mixed-methods design, including prevalence estimates with 95% confidence intervals (CI) and qualitative thematic coding of write-in responses for respondents who said “it depends.”

Quantitative analysis was conducted using Stata version 15.1 (StataCorp LP, College Station, TX) and the SVY suite of commands. All estimates are weighted to be representative of the adult population of California using a weight variable provided by Ipsos. The weight variable combines a pre-sample weight, which adjusts for the probability of selection into KnowledgePanel and aligns the sample to geodemographic benchmarks for the US population, and a study-specific post-stratification weight, which accounts for mis-representation of key demographics between survey respondents and the adult population of California and for survey non-response.

For qualitative analysis of write-in responses, two authors (RP and NKW) served as coders. Coders independently read a sample (10%) of responses and summarized key components and common elements to develop a coding instrument. Coders met to discuss codes and organize codes under superordinate themes. Coders iteratively revised the codebook throughout the coding process, adding emergent codes for relevant elements that appeared in the data. After meeting to confirm completeness of the codebook and contextual authenticity, the coders double-coded all open-ended responses to each question and compared coding. The coders discussed all discrepancies and came to agreement on the final code applied.

When a write-in response included several ideas, the responses were coded with multiple codes. Write-in responses included a maximum of four codes; most responses involved one or two codes (93.8% for guns in their homes and 91.1% for guns in all homes in their neighborhoods).

Coding was conducted in Microsoft Excel (v.16.46) and counts and weighted proportions were calculated using Stata (v.15.1) and SVY commands.

## Results

### Sample

**Fig 1:**
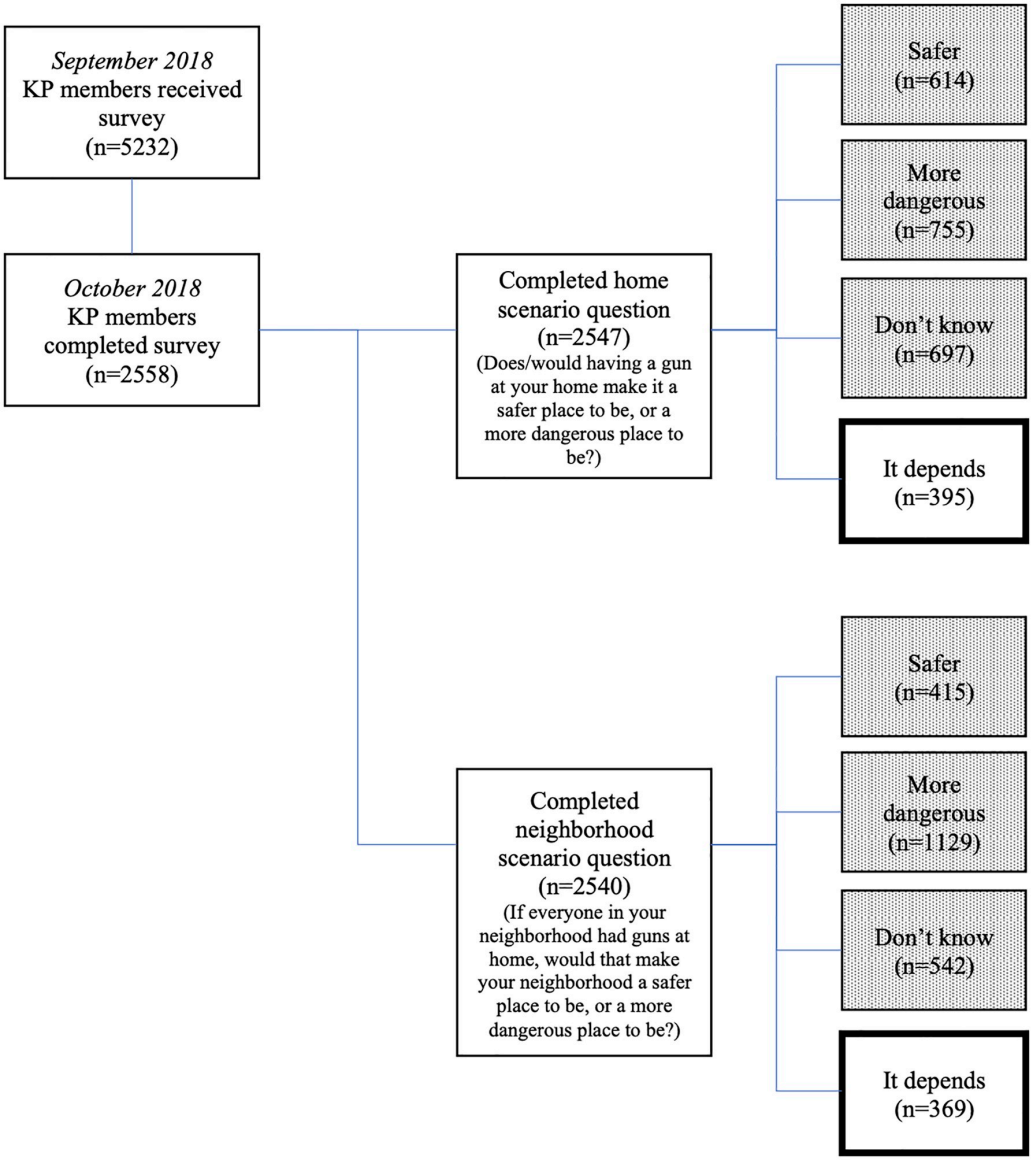
Recruitment and participation of sample. Note: Counts are unweighted. Dotted boxes denote groups that are not shown in these analyses. Groups detailed in thick outlined boxes are included in these analyses.

A total of 2,558 (49%) invited panel members completed CSaWS; 2,547 respondents completed the home scenario question and 2,540 completed the neighborhood scenario question (Fig 1). Respondents were 52.3% (95% CI 49.2% to 55.4%) female and 42.7% (95% CI 39.8% to 45.6%) non-Latinx white (Tables 1 and 2). Mean respondent age was 47.8 (SD = 17.0). One in seven respondents owned firearms (14.4%, 95% CI 12.5% to 16.4%) and 10.5% (95% CI 8.7% to 12.6%) lived in homes with firearms but did not personally own them. One in six respondents (16.9%, 95% CI 14.7% to 19.4%) reported “it depends” when asked about the home scenario (Table 1), and a similar percentage (16.2%, 95% CI 14.0% to 18.8%) said “it depends” when asked about the neighborhood scenario (Table 2).

**Table 1:** (Does/Would) having a gun at your home make it a safer place to be, or a more dangerous place to be?, by demographics and gun ownership status.

	Safer	More dangerous	It depends	Don’t know	Total	
	Weighted % (95% CI)	Weighted % (95% CI)	Weighted % (95% CI)	Weighted % (95% CI)	Weighted % (95% CI)	Unweighted N
Total	25.3 (22.7 to 28.0)	28.8 (26.1 to 31.6)	16.9 (14.7 to 19.4)	29.1 (26.3 to 32.0)	-	2547
Age						
18–29	20.7 (15.0 to 27.8)	15.3 (11.1 to 20.8)	18.5 (12.5 to 26.6)	17.2 (12.2 to 23.5)	17.8 (15.0 to 20.9)	158
30–44	21.1 (16.4 to 26.7)	32.4 (27.1 to 38.2)	29.3 (22.8 to 36.8)	27.5 (22.3 to 33.5)	27.6 (24.8 to 30.6)	477
45–59	30.0 (25.0 to 35.5)	23.9 (19.4 to 29.0)	30.1 (23.7 to 37.3)	26.2 (21.4 to 31.6)	27.1 (24.5 to 29.9)	676
60+	28.2 (24.1 to 32.7)	28.3 (24.1 to 33.0)	22.1 (17.4 to 27.7)	29.1 (24.8 to 33.9)	27.5 (25.2 to 29.9)	1,236
Gender						
Male	58.2 (52.3 to 63.9)	44.6 (39.0 to 50.4)	47.8 (40.3 to 55.4)	41.5 (35.7 to 47.7)	47.7 (44.6 to 50.8)	1,082
Female	41.8 (36.1 to 47.7)	55.4 (49.6 to 61.0)	52.2 (44.6 to 59.7)	58.5 (52.3 to 64.3)	52.3 (49.2 to 55.4)	1,465
Race-Ethnicity						
White, Non-Latinx	54.8 (48.6 to 60.9)	36.3 (31.6 to 41.4)	43.6 (36.5 to 50.9)	37.9 (32.8 to 43.3)	42.7 (39.8 to 45.6)	1,439
Black, Non-Latinx	5.0 (3.0 to 8.2)	4.9 (3.1 to 7.6)	6.1 (3.1 to 11.7)	6.2 (3.9 to 9.7)	5.5 (4.3 to 7.1)	121
Other*, Non-Latinx	8.3 (5.6 to 12.2)	19.4 (14.9 to 24.8)	22.2 (15.9 to 30.1)	16.1 (11.8 to 21.4)	16.1 (13.7 to 18.8)	191
Latinx	28.7 (22.9 to 35.3)	37.7 (32.0 to 43.8)	25.4 (19.1 to 33.0)	37.4 (31.4 to 43.9)	33.3 (30.2 to 36.6)	739
2+ Races, Non-Latinx	3.2 (1.6 to 6.4)	1.6 (0.9 to 2.9)	2.7 (1.4 to 5.4)	2.4 (1.2 to 4.9)	2.4 (1.7 to 3.5)	57
Urban/Rural Status						
Urban	87.1 (82.9 to 90.5)	93.4 (89.9 to 95.8)	89.5 (84.0 to 93.3)	94.3 (91.7 to 96.1)	91.4 (89.7 to 92.9)	2,263
Sub-urban or rural	12.9 (9.5 to 17.1)	6.6 (4.2 to 10.1)	10.5 (6.7 to 16.0)	5.7 (3.9 to 8.3)	8.6 (7.1 to 10.3)	261
Gun ownership status						
No guns in home	41.8 (35.7 to 48.1)	95.7 (93.4 to 97.3)	71.4 (64.3 to 77.6)	81.8 (77.1 to 85.7)	74.1 (71.3 to 76.7)	1,794
Gun owner	36.4 (30.9 to 42.3)	2.3 (1.2 to 4.2)	18.8 (13.4 to 25.7)	6.8 (4.9 to 9.4)	14.9 (13.0 to 17.1)	425
Non-owner in gun household	21.8 (16.7 to 28.1)	2.0 (1.0 to 3.7)	9.8 (6.9 to 13.9)	11.4 (8.1 to 15.7)	11.0 (9.1 to 13.2)	242

11 respondents refused to answer and are excluded from this table.

*”Other” race includes respondents who selected American Indian or Alaska Native, Asian, Native Hawaiian or Pacific Islander, or “some other race”.

Note. All percentages are weighted and all counts are unweighted.

**Table 2:** If everyone in your neighborhood had guns at home, would that make your neighborhood a safer place to be, or a more dangerous place to be?, by demographics and gun ownership status.

	Safer	More dangerous	It depends	Don’t know	Total	
	Weighted % (95% CI)	Weighted % (95% CI)	Weighted % (95% CI)	Weighted % (95% CI)	Weighted % (95% CI)	Unweighted N
Total	16.9 (14.8 to 19.2)	44.5 (41.4 to 47.6)	16.2 (14.0 to 18.8)	22.4 (19.9 to 25.1)	-	2540
Age						
18–29	10.8 (6.1 to 18.5)	16.4 (12.8 to 20.9)	24.8 (17.3 to 34.3)	19.5 (13.7 to 26.8)	17.5 (14.8 to 20.7)	156
30–44	29.1 (22.6 to 36.6)	29.8 (25.5 to 34.5)	27.4 (20.6 to 35.5)	22.7 (17.6 to 28.6)	27.7 (24.9 to 30.7)	475
45–59	31.1 (25.0 to 37.8)	27.3 (23.3 to 31.8)	22.0 (17.0 to 28.0)	28.0 (22.7 to 34.0)	27.3 (24.6 to 30.0)	674
60+	29 (24.2 to 34.3)	26.4 (23.0 to 30.0)	25.7 (20.2 to 32.1)	29.9 (25.1 to 35.2)	27.5 (25.2 to 29.9)	1,235
Gender						
Male	64.1 (57.3 to 70.5)	44.0 (39.3 to 48.8)	51.2 (43.1 to 59.1)	41.1 (34.8 to 47.8)	47.9 (44.8 to 51.0)	1,081
Female	35.9 (29.5 to 42.7)	56.0 (51.2 to 60.7)	48.8 (40.9 to 56.9)	58.9 (52.2 to 65.2)	52.1 (49.0 to 55.2)	1,459
Race-Ethnicity						
White, Non-Latinx	61.5 (54.2 to 68.4)	36.2 (32.3 to 40.3)	43.4 (35.8 to 51.2)	41.6 (35.7 to 47.8)	42.9 (39.9 to 45.8)	1,440
Black, Non-Latinx	1.6 (0.7 to 3.9)	4.7 (3.2 to 7.0)	7.5 (4.3 to 12.6)	8.5 (5.3 to 13.4)	5.5 (4.3 to 7.1)	121
Other*, Non-Latinx	10.4 (6.6 to 16.0)	19 (15.2 to 23.5)	13.8 (8.8 to 20.8)	16.6 (12.1 to 22.4)	16.2 (13.8 to 18.8)	191
Latinx	24.4 (18.3 to 31.7)	38.4 (33.6 to 43.4)	31.7 (24.1 to 40.5)	29.8 (23.8 to 36.7)	33.0 (29.9 to 36.3)	731
2+ Races, Non-Latinx	2.1 (1.0 to 4.3)	1.7 (1.0 to 2.7)	3.7 (1.5 to 8.7)	3.4 (1.7 to 6.7)	2.5 (1.7 to 3.5)	57
Urban/Rural Status						
Urban	86.4 (80.9 to 90.4)	93.5 (91.0 to 95.3)	90.3 (84.9 to 94.0)	91.8 (87.8 to 94.6)	91.4 (89.6 to 92.9)	2,259
Sub-urban or rural	13.6 (9.6 to 19.1)	6.5 (4.7 to 9.0)	9.7 (6.0 to 15.1)	8.2 (5.4 to 12.2)	8.6 (7.1 to 10.4)	260
Gun ownership status						
No guns in home	39.2 (32.4 to 46.5)	89.9 (87.0 to 92.2)	63.5 (55.5 to 70.8)	75.4 (69.6 to 80.5)	74.1 (71.3 to 76.7)	1,788
Gun owner	43.4 (36.3 to 50.7)	4.5 (2.9 to 6.7)	19.4 (14.3 to 25.7)	11.9 (8.6 to 16.2)	14.9 (13.0 to 17.1)	425
Non-owner in gun household	17.4 (11.7 to 25.0)	5.7 (4.0 to 7.9)	17.1 (11.7 to 24.4)	12.7 (8.9 to 17.8)	11.0 (9.2 to 13.2)	242

18 respondents refused to answer and are excluded from this table.

*”Other” race includes respondents who selected American Indian or Alaska Native, Asian, Native Hawaiian or Pacific Islander, or “some other race.”

Note. All percentages are weighted and all counts are unweighted.

For both scenarios, the proportions of respondents who said “it depends” did not vary substantially by age, gender, race-ethnicity, or urban-rural residence. For the home scenario, responses differed by gun ownership status: gun owners disproportionately said “safer” or “it depends” compared to other responses.

### Themes: What does it depend on?

Five primary themes emerged from the factors on which respondents said firearm risk perceptions depended: (1) characteristics of gun owners: “who owns them;” (2) at-risk person in the home: “I wouldn’t want him to hurt himself;” (3) storage and access: “how accessible the guns are;” (4) the neighborhood: “how safe the overall neighborhood is;” and (5) intentions for gun use: “what they intend to use it for.” Characteristics of gun owners had two subthemes: (a) knowledge and proficiency: “if the gun owner is trained properly;” and (b) temperament and mental health: “how stable the gun owners are.” Intentions for gun use included one subtheme: (c) self- and home- protection: “if used to protect yourself from intruders.” Illustrative quotes, as well as their corresponding codes and themes, are in Table 3.

**Table 3:** Themes, codes, and sample of excerpts for factors on which perceived safety of guns in the home depends, by home and neighborhood scenarios.

Themes*	Codes	Example excerpts: the home scenario	Example excerpts: the neighborhood scenario
**Characteristics of gun owners**			
Knowledge and proficiency	Handling/safety trained; confidence or proficiency with firearm use	"If I know how to use it"	"Who they are and the knowledge, experience and training of the gun owner"
		"On whether everyone in the house was completely trained in the use of the gun."	"If everyone was taught how to use them and if they used them properly, then the neighborhood would be safer. However, if some of them didn’t really know how to handle them properly, then things could be more dangerous."
		"If the person handling it has been properly trained."	"Gun safety knowledge of owner"
Temperament and mental health	Responsible firearm owner; “law abiding”; dangerous/criminal activity; mental health; trustworthiness; substance use; temperament	"On my mental health"	"On how responsible the gun owner was."
		"How I use it and my emotions."	"If the ones that have a gun, are they mentally stable to have a gun, and how do they handle conflict, because that determines if the neighborhood is more in dangerous or safe"
		"If you have a bad temper"	"The mental state, maturity, skill, and criminal history of my neighborhood."
		"Responsibility of the owner of the gun"	"Depends on the mindset of the person owning the gun."
**At-risk person in the home**	Children; others	"It depends if there are children"	"if they are put up and stored away from children and used only when there is an emergency"
		"Who is in the household and anyone who is feeling depressed."	"If there are children in the house, then there shouldn’t be any guns. I personally believe that kids get curious and there *(sic)* too stupid nowadays to have any weapons in the house. That’s how accidents start happening."
**Storage and access**	Firearms stored safely; accessible to those who shouldn’t have access; quickly accessible; who knows about the firearms in the home	"How accessible the guns are—if they are locked in the home in a safe or not and who has access."	"It depends on how the other gun owners store their guns and ammunition"
		"On how fast I can get to gun if needed."	"How safe the gun owner stores and uses the gun."
		"it depends on who knows the gun is in the house and if it is ever talked about"	"If they are responsible to keep their guns in a safe and not use it unless they have [to]."
**The neighborhood**	Individual threats (e.g., threats from animals, law enforcement response time); environmental threats (e.g., safety of the neighborhood, government takeover, mistrust of neighbors)	"If I owned a gun and there was consistent violence in my neighborhood, I might feel safer with a gun."	"If all of a sudden there is crime in our neighborhood."
		"I am more concerned about protection from the wild animals in my area than humans."	“Our neighborhood is safe. If that was to change, [then] I guess a gun would be needed.”
		"How fast the police response time is. Most people [don’t] need them."	"It depends on the neighborhoods and safety of the city and the people"
**Intentions for gun use**	Reasons for ownership/use	"Why/how you need to use it."	"On whether there would be a need to use it."
		"It would depend on whether there were feelings/senses of apparent danger!!!"	"If everyone was trigger happy and used guns for [every] situation."
Self- or home protection	Mention of having/needing/using a gun for self- or home- protection	"If for example our home gets broken into, if it could be reached for in time and to deter the burglar."	"On whether those guns were kept simply for personal safety and on no other possible intentions"
		"If I were to be the victim of a crime within my home"	"It depends on whether everyone only used guns for self-defense, not murder."
		"If a person breaking in finds and takes the gun, then more dangerous. If the owner of the gun gets it first, then safer."	"It depends on how they are used. If used to protect yourself from intruders that is the purpose otherwise they are dangerous."

*Primary themes are bolded.

**Fig 2:**
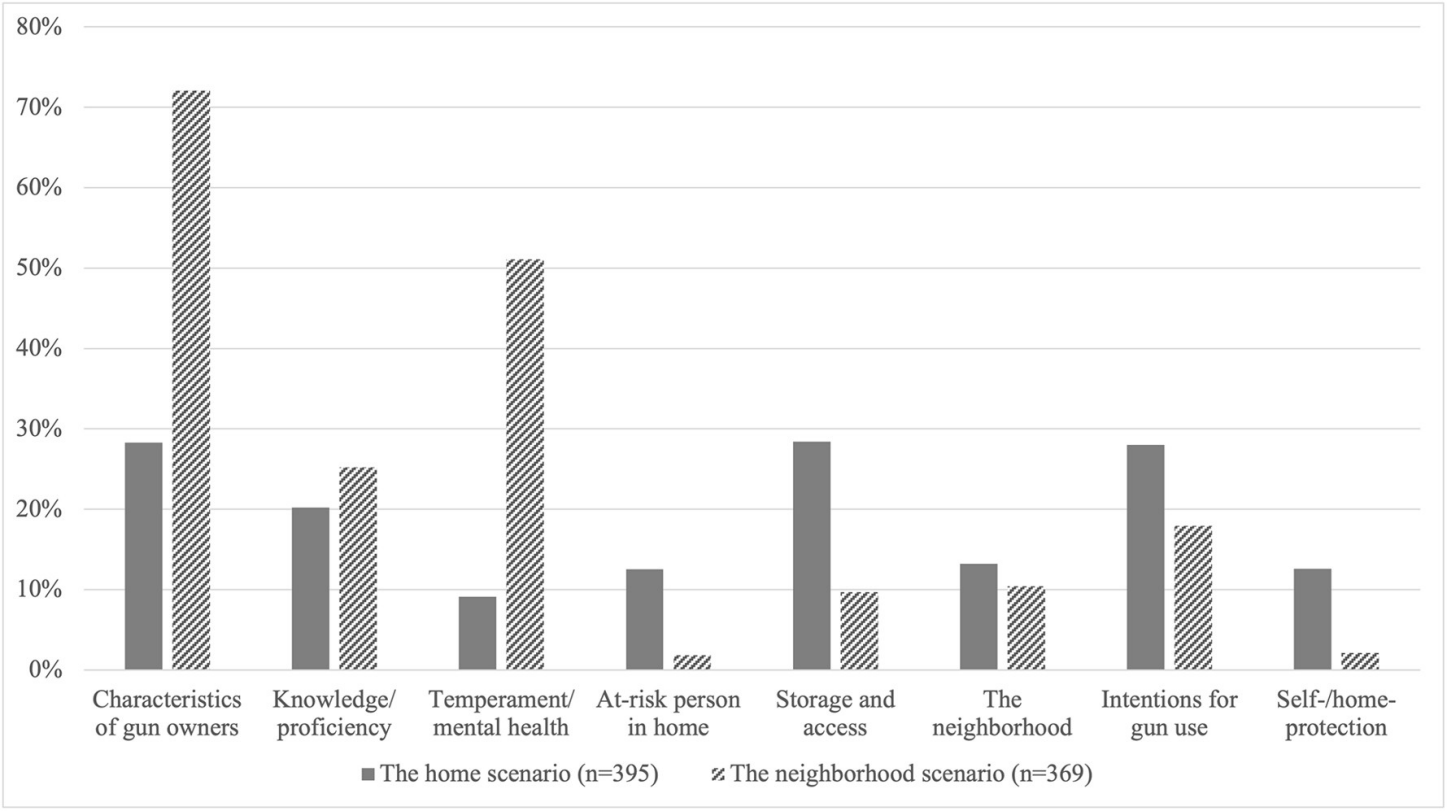
Factors on which perceptions of safety depend: The home scenario vs the neighborhood scenario.

Overall, respondents more often cited intentions for gun use and storage and access in response to the home scenario compared with the neighborhood scenario and more often reported characteristics of gun owners, especially temperament and mental health, in response to the neighborhood scenario compared with the home scenario (Fig 2).

#### Theme 1—Characteristics of gun owners: “Who owns them”

Respondents most often conditioned their responses on the characteristics of gun owners in both scenarios, and especially so in the neighborhood scenario (72.1%, 95% CI 63.4% to 79.3% vs 28.3%, 95% CI 21.9% to 35.7% for the home scenario) (Tables 4 and 5). In particular, respondents indicated that safety or dangerousness depended on whether gun owners were knowledgeable about and proficient in using guns safely. Others perceived that temperament and mental health characteristics of gun owners, including owners’ perceived involvement in illegal or dangerous activities (e.g., substance misuse, crime, gangs), played a role. Knowledge and proficiency were more often noted for the home scenario, whereas temperament and mental health characteristics were more often cited with respect to the neighborhood scenario. Among firearm ownership status groups, temperament and mental health characteristics were cited least often by gun owners in both scenarios.

**Table 4:** Percentage of respondents who reported “it depends” whether guns make homes safer or more dangerous and factors on which it depends, by firearm ownership status. The home scenario.

	No guns in household	Gun owner	Non-owner in gun household	TOTAL	
	Weighted % (95% CI)	Weighted % (95% CI)	Weighted % (95% CI)	Weighted % (95% CI)	Unweighted N
TOTAL^a^	16.5 (13.9 to 19.5)	21.6 (15.6 to 29.0)	15.4 (10.7 to 21.6)	17.2 (14.9 to 19.7)	395
Themes^b^					
Characteristics of gun owners	29.5 (21.6 to 39.0)	24.9 (13.8 to 40.9)	26.4 (14.0 to 44.1)	28.3 (21.9 to 35.7)	108
Knowledge/proficiency	18.6 (12.1 to 27.6)	24.1 (13.1 to 40.1)	23.0 (11.0 to 41.8)	20.2 (14.7 to 27.1)	81
Temperament/mental health	11.9 (6.4 to 21.2)	1.0 (0.1 to 7.1)	6.3 (2.0 to 18.4)	9.1 (5.1 15.6)	24
At-risk person in home	14.6 (9.5 to 21.9)	4.4 (1.6 to 11.5)	13.4 (6.0 to 27.2)	12.5 (8.6 to 17.8)	54
Storage and access	28.3 (21.0 to 36.8)	26.1 (13.6 to 44.2)	33.7 (18.7 to 53.0)	28.4 (22.3 to 35.5)	128
The neighborhood	15.8 (9.7 to 24.5)	5.4 (2.1 to 13.2)	10.9 (3.2 to 30.9)	13.2 (8.7 to 19.7)	46
Intentions for gun use	27.5 (19.3 to 37.5)	18.9 (10.3 32.2)	48.4 (31.3 to 65.8)	28.0 (21.5 to 35.7)	106
Self- or home-protection	12.4 (7.4 to 20.1)	9.4 (4.2 to 19.6)	19.3 (10.3 to 33.1)	12.6 (8.5 to 18.1)	53
Other	7.5 (3.5 to 15.5)	8.4 (2.9 to 22.0)	8.7 (2.1 to 29.9)	7.8 (4.4 to 13.6)	22

^a^. The “Total” row shows the percentages of *all* respondents (n = 2558) who said “it depends” to the home scenario question, by gun ownership status.

^b^. The “Themes” rows show the percentages of respondents who report each theme among those (n = 395) who said “it depends” to the home scenario question, by firearm ownership status.

Note: Responses are not mutually exclusive. 28 respondents who said "it depends" did not provide a write-in answer and 9 wrote "neither." All percentages are weighted and all counts are unweighted.

**Table 5:** Percentage of respondents who reported “it depends” whether guns make homes safer or more dangerous and factors on which it depends, by firearm ownership status. The neighborhood scenario.

	No guns in household	Gun owner	Non-owner in gun household	Total	
	Weighted % (95% CI)	Weighted % (95% CI)	Weighted % (95% CI)	Weighted % (95% CI)	Unweighted N
TOTAL^a^	14.2 (11.6 to 17.2)	21.5 (16.2 to 28.0)	25.8 (18.0 to 35.6)	16.6 (14.3 to 19.2)	369
Themes^b^					
Characteristics of gun owners	77.1 (65.9 to 85.5)	49.8 (34.6 to 65.1)	80.8 (58.7 to 92.6)	72.1 (63.4 to 79.3)	276
Knowledge/proficiency	27.4 (18.7 to 38.3)	26.2 (16.1 to 39.7)	14.4 (6.5 to 29.1)	25.2 (18.8 to 32.9)	108
Temperament/mental health	55.3 (43.1 to 66.9)	24.9 (14.8 to 38.8)	68.5 (44.8 to 85.4)	51.1 (41.8 to 60.2)	148
At-risk person in home	2.0 (0.3 to 12.8)	2.8 (0.4 to 17.5)	0	1.8 (0.4 to 7.5)	2
Storage and access	11.7 (6.1 to 21.3)	7.3 (2.9 to 17.3)	5.6 (1.6 to 17.5)	9.7 (5.7 to 15.9)	31
The neighborhood	10.8 (5.3 to 20.8)	16.5 (7.8 to 31.4)	1.7 (0.6 to 5.2)	10.4 (6.2 to 16.9)	33
Intentions for gun use	17.2 (9.8 to 28.3)	23.2 (10.5 to 43.8)	13.9 (4.0 to 38.5)	17.9 (11.7 to 26.3)	59
Self- or home-protection	2.4 (0.7 to 8.4)	2.5 (0.4 to 15.8)	0.7 (0.2 to 3.1)	2.1 (0.8 to 5.8)	8
Other	2.7 (1.2 to 6.2)	13.6 (4.8 to 32.9)	0.9 (0.1 to 6.5)	4.7 (2.3 to 9.4)	13

^a^. The “Total” row shows the percentages of *all* respondents (n = 2558) who said “it depends” to the neighborhood scenario question, by gun ownership status.

^b^. The “Themes” rows show the percentages of respondents who report each theme among those (n = 369) who said “it depends” to the neighborhood scenario question, by gun ownership status.

Note: Responses are not mutually exclusive. 22 respondents who said "it depends" did not provide a write-in answer. All percentages are weighted and all counts are unweighted.

#### Theme 2—At-risk person in the home: “I wouldn’t want him to hurt himself”

Respondents also noted the presence of household members who are at elevated risk for firearm-related harm as affecting perceived safety of guns in the home. This included children in the home and household members with mental health or cognitive problems.

For example, one respondent explained: "I feel it would be safer if someone were to break in but honestly I would worry about [my] spouse. To me he always seems depressed. I wouldn’t want him to hurt himself, my children or me." Another wrote: "If the gun was safely put away, it would make the home a safer place; however, I have a son with Autism, and the thought of him finding the gun scares me. He may think it’s a toy and wouldn’t understand the danger of handling a gun."

At-risk people in the home were mentioned by 12.5% (95% CI 8.6% to 17.8%) of respondents—including slightly higher percentages of non-owners in gun and non-gun households (13.4%, 95% CI 6.0% to 27.2%, and 14.6%, 95% CI 9.5% to 21.9%, respectively)—when questioned about guns in their own homes. Two-thirds of these responses (63.5%, 95% CI 44.1% to 79.1%) specifically referenced the presence of children. At-risk people in the home was rarely mentioned in the context of the neighborhood scenario.

#### Theme 3—Storage and access: “How accessible the guns are”

More than one in four respondents (28.4%, 95% CI 22.3% to 35.5%), regardless of firearm ownership status, said the safety or dangerousness of guns in their homes depended on gun storage and access. This included factors such as who could access them, whether guns were stored locked up, and how quickly the respondent could access the gun “if needed.” Storage and access were mentioned by 9.7% (95% CI 5.7% to 15.9%) of respondents in the neighborhood scenario.

#### Theme 4—Neighborhood safety: “How safe the overall neighborhood is”

Slightly more than one in ten respondents reported that the safety of the neighborhood and perceived threats affected firearm-related safety in each scenario:13.2% (95% CI 8.7% to 19.7%) for the home scenario and 10.4% (95% CI 6.2% to 16.9%) for the neighborhood scenario. Perceived threats from people and animals, levels of neighborhood crime and violence, and local law enforcement response times were mentioned. For the home scenario, non-owners in gun and non-gun households more often mentioned these neighborhood safety-related stipulations and gun owners more often cited them for the neighborhood scenario.

#### Theme 5—Intentions or circumstances for gun use: “What they intend to use it for”

Perceived safety of guns in the home depended on the user’s intentions, specifically reasons for gun ownership, for nearly one in three (28.0%, 95% CI 21.5% to 35.7%) respondents in the home scenario and one in five (17.9%, 95% CI 11.7% to 26.3%) respondents in the neighborhood scenario. Self- and home-protection were mentioned often in the home scenario: 44.8% (95% CI 31.0% to 59.5%) specifically noted that safety depended on whether the gun would be used for self-protection.

## Discussion

In this state-representative, mixed-methods survey study of California adults, respondents reported whether guns in their homes and guns in all homes in their neighborhoods make them safer places to be, more dangerous places to be, or whether “it depends.” In both the home and neighborhood scenarios, approximately 1 in 6 respondents overall and 1 in 5 gun owners said “it depends” whether guns make homes safer or more dangerous. These percentages are lower than prior national estimates, such as the 56% estimate among gun owners in the 2019 NFS [8], though our survey included a “don’t know” response option endorsed by 29% and 22% of respondents, respectively, for the home and neighborhood scenarios.

When those who reported “it depends” were asked to elaborate on what their perceptions of risk depended, five primary themes emerged, which varied by scenario and by firearm ownership status. The importance of owners being knowledgeable about and proficient in using guns safely was most commonly reported, and was raised more often among gun owners than non-owners, regardless of scenario. This suggests that gun owners could be receptive to injury prevention efforts in which individuals living in homes with guns receive training on safe firearm use and storage.

Respondents, especially those who did not own firearms, also specified other characteristics of gun owners, including temperament and mental health status (including “responsible” gun ownership, mental or emotional health, and problems with substance use), as conditioning their perceptions of risk. This suggests that a small but notable percentage of respondents recognize that access to household firearms can elevate risk when someone is experiencing an emotional crisis or behaving dangerously. This finding may provide an important foundation on which to build momentum for targeted, risk-based approaches to preventing firearm violence before it occurs. Such approaches include temporary, voluntary transfer of firearms out of the home when someone is suicidal (i.e., lethal means reduction). They also include extreme risk protection orders (ERPOs), a legal tool available in a growing number of states that allows for temporary removal of guns and limits the ability to purchase them for individuals that a judge has deemed to be at significant risk of firearm-related harm.

More than 1 in 4 of our respondents who said “it depends,” regardless of firearm ownership status, considered gun storage and access as a factor influencing safety when guns were in their own home. As firearm storage that reduces access to at-risk persons may reduce gun-related injuries, further public health interventions promoting secure firearm storage are warranted. Such interventions could benefit from directly addressing misperceptions about the benefits and risks of household gun ownership that are counter to epidemiologic evidence, or the so-called “protection paradox” [15].

Given substantial evidence that a firearm in the home increases risk for, in particular, suicide and unintentional injury for all members of the household [1, 2, 16], storing firearms such that they are not accessible to unauthorized users or those at risk, such as children or people with suicidal ideation or intent, is widely recommended to reduce risk of injury and death [16–18]. However, just 12% of respondents who said “it depends,” including 4% of owners and 13% of non-owners in gun households, conditioned their responses on whether there was a person at risk for injury in the home. This finding suggests an opportunity for improved messaging about risk factors for firearm-related harm. Past work has found, for example, that both gun owners and non-owners in gun households are particularly receptive to gun safety conversations with health professionals when risk for firearm-related harm (e.g., there is access to firearms and someone is having thoughts of suicide, or lives with children or teens) has been established [19]. Collaborative work between gun owners and injury prevention experts may help improve our understanding of barriers to risk reduction strategies and in the development of informed, realistic interventions.

## Limitations

As with all survey research, our results are subject to non-response, recall, and social desirability biases. Participants in our study may be different than panel members who chose not to participate (e.g., non-responders were more often female, younger, Latinx, had fewer years of education, had lower income, and lived in homes with children). Our survey was among respondents in California, which has relatively restrictive firearm policies and relatively low rates of firearm ownership, possibly limiting the generalizability of these results. The relatively small sample size and subsample of gun-owning respondents limits this work to descriptive statistics and constrains our ability to conduct further subgroup analyses.

Additionally, the survey questions limit our interpretation and comparison of the responses to questions about the home and neighborhood scenarios. It is possible that in the neighborhood scenario, respondents are not thinking of safety in their neighbors’ homes but rather of safety in the public spaces in their neighborhood (i.e., about the impact of neighbors’ guns on respondents themselves). We chose this framing, however, to maintain relevance to the respondent.

## Conclusion

Perceived risks associated with household firearms varied by firearm ownership status and whether respondents were considering guns in their own homes or all homes in their neighborhoods. A substantial proportion of respondents said the extent to which guns make homes safer or more dangerous depended on gun owner characteristics such as knowledge of and proficiency with firearms, temperament and mental health, firearm storage practices, and other considerations. Future research should further examine factors that affect gun owners’ perceptions of safety in order to inform the development of interventions that consider the ways in which individuals characterize risk when there are guns in the home.

## Supporting information

S1 Appendix Question text and response options as presented in the 2018 California Safety and Wellbeing Survey (CSaWS) and included in analyses for “‘What does it depend on?’: Perceptions of safety related to firearms in homes and neighborhoods”. (DOCX)

S1 Table Data for Fig 2 (percentages of respondents who cited key themes and subthemes on which perceived safety of guns in the home depends). (DOCX)

S1 Data (CSV)
