# Supplementary material for: “What does it depend on?”: Perceptions of safety related to firearms in homes and neighborhoods
Source: PLoS One. 2021 Dec 29;16(12):e0261038. doi: 10.1371/journal.pone.0261038 (PMC8716056; doi:10.1371/journal.pone.0261038)
Supplement: S1 Appendix — (DOCX) [file pone.0261038.s001.docx]

**S1 Appendix.** Question text and response options as presented in 2018 California Safety and Wellbeing Survey (CSaWS) included in analysis for “’What does it depend on?’: perceptions of safety related to firearms in homes and neighborhoods.”

Note: Respondent demographic information is collected by Ipsos upon recruitment into the panel and provided with the survey data. This includes information on respondent age, gender, race/ethnicity, and urbanicity of residence.

Do you or does anyone else you live with currently own any type of gun?

Yes

No

Don’t know

Do you personally own a gun?

Yes

No

Does/would having a gun at your home make it a safer place to be, or a more dangerous place to be?

Safer

More dangerous

It depends

Don’t know

[If chose “it depends”] What does it depend on?

[Write-in text option]

If everyone in your neighborhood had guns at home, would that make your neighborhood a safer place to be, or a more dangerous place to be?

Safer

More dangerous

It depends

Don’t know

[If chose “it depends”] What does it depend on?

[Write-in text option]
