# Supplementary material for: “What does it depend on?”: Perceptions of safety related to firearms in homes and neighborhoods
Source: PLoS One. 2021 Dec 29;16(12):e0261038. doi: 10.1371/journal.pone.0261038 (PMC8716056; doi:10.1371/journal.pone.0261038)
Supplement: S1 Table — (DOCX) [file pone.0261038.s002.docx]

**S1 Table.** Data for Figure 2 (Percentages of respondents who cited key themes and subthemes on which firearm-related safety depends)

|  | The home scenario (n=395) | The neighborhood scenario (n=369) |
| --- | --- | --- |
|  | % (95% CI) | % (95% CI) |
| Characteristics of gun owners | 28.3 (21.9,35.7) | 72.1 (63.4, 79.3) |
| Knowledge/proficiency | 20.2 (14.7,27.1) | 25.2 (18.8,32.9) |
| Temperament/mental health | 9.1 (5.1 15.6) | 51.1 (41.8,60.2) |
| At-risk person in home | 12.5 (8.6,17.8) | 1.8 (0.4, 7.5) |
| Storage and access | 28.4 (22.3, 35.5) | 9.7 (5.7,15.9) |
| The neighborhood | 13.2 (8.7, 19.7) | 10.4 (6.2,16.9) |
| Intentions for gun use | 28.0 (21.5, 35.7) | 17.9 (11.7,26.3) |
| Self- or home-protection | 12.6 (8.5, 18.1) | 2.1 (0.8, 5.8) |
| Note. All percentages are weighted and all counts are unweighted. | | |
